# Neurocognitive robot-assisted rehabilitation of hand function: a randomized control trial on motor recovery in subacute stroke

**DOI:** 10.1186/s12984-020-00746-7

**Published:** 2020-08-24

**Authors:** Raffaele Ranzani, Olivier Lambercy, Jean-Claude Metzger, Antonella Califfi, Stefania Regazzi, Daria Dinacci, Claudio Petrillo, Paolo Rossi, Fabio M. Conti, Roger Gassert

**Affiliations:** 1 Rehabilitation Engineering Laboratory, Institute of Robotics and Intelligent Systems, Department of Health Sciences and Technology, BAA Lengghalde 5, 8008 Zurich, Switzerland; 2 Clinica Hildebrand Centro di Riabilitazione Brissago, Via Crodolo, 6614 Brissago, Switzerland

**Keywords:** Neurorehabilitation, Stroke, Hand, Robotics, Haptics

## Abstract

**Background:**

Hand function is often impaired after stroke, strongly affecting the ability to perform daily activities. Upper limb robotic devices have been developed to complement rehabilitation therapy offered to persons who suffered a stroke, but they rarely focus on the training of hand sensorimotor function. The primary goal of this study was to evaluate whether robot-assisted therapy of hand function following a neurocognitive approach (i.e., combining motor training with somatosensory and cognitive tasks) produces an equivalent decrease in upper limb motor impairment compared to dose-matched conventional neurocognitive therapy, when embedded in the rehabilitation program of inpatients in the subacute stage after stroke.

**Methods:**

A parallel-group, randomized controlled trial was conducted on subjects with subacute stroke receiving either conventional or robot-assisted neurocognitive hand therapy using a haptic device. Therapy was provided for 15, 45-min sessions over four weeks, nested within the standard therapy program. Primary outcome was the change from baseline in the upper extremity part of the Fugl-Meyer Assessment (FMA-UE) after the intervention, which was compared between groups using equivalence testing. Secondary outcome measures included upper limb motor, sensory and cognitive assessments, delivered therapy dose, as well as questionnaires on user technology acceptance.

**Results:**

Thirty-three participants with stroke were enrolled. 14 subjects in the robot-assisted and 13 subjects in the conventional therapy group completed the study. At the end of intervention, week 8 and week 32, the robot-assisted/conventional therapy group improved by 7.14/6.85, 7.79/7.31, and 8.64/8.08 points on the FMA-UE, respectively, establishing that motor recovery in the robot-assisted group is non-inferior to that in the control group.

**Conclusions:**

Neurocognitive robot-assisted therapy of hand function allows for a non-inferior motor recovery compared to conventional dose-matched neurocognitive therapy when performed during inpatient rehabilitation in the subacute stage. This allows the early familiarization of subjects with stroke to the use of such technologies, as a first step towards minimal therapist supervision in the clinic, or directly at home after hospital discharge, to help increase the dose of hand therapy for persons with stroke.

**Trial registration:**

EUDAMED database (CIV-13-02-009921), clinicaltrials.gov (NCT02096445). Registered 26 March 2014 – Retrospectively registered, https://clinicaltrials.gov/ct2/show/NCT02096445

## Background

Upper-limb robot-assisted therapy has been established as a safe and feasible treatment to complement rehabilitation after neurological injury, such as stroke [1]. Robots can precisely control the interaction with the user (e.g., supporting or resisting in an assist-as-needed manner) and render virtual environments both visually and mechanically, making them ideal tools for sensorimotor training, providing engaging and challenging therapy [2, 3]. Over the past two decades, several robotic devices to train the proximal upper extremity [4] were developed and clinically evaluated, achieving outcomes comparable to dose-matched conventional therapy [1–3, 5–10].

However, distal arm function is essential for the execution of activities of daily living (e.g., eating, dressing) and is often severely impaired after stroke [11], with low probability of regaining its full functional use [12]. Several studies have shown that functional motor training at the level of the hand with robotic devices can be beneficial and positively translate into recovery of proximal arm function [13, 14]. Despite recent investigations to develop novel robots to train hand function [9, 15, 16], only few systems took advantage of the haptic rendering capabilities of robots to support somatosensory training, nor evaluated this in clinical trials. As such, most systems for robot-assisted therapy developed to date focus on movement practice without incorporating an established therapy concept adapted to the capabilities of the respective technology.

In this work, the clinical equivalence of sensorimotor, robot-assisted rehabilitation of hand function is investigated within a four-week randomized controlled trial (RCT) on subacute stroke participants. The neurocognitive rehabilitation method proposed by Perfetti [17] was selected as reference therapy approach. It focuses on the training of sensorimotor functions as well as cognition, which is fundamental during functional interactions between body and environment (e.g., information perception, as well as elaboration, selection and execution of motor plans) [18–20]. Because of the relevance of the cognitive processing of sensory inputs, this approach is particularly interesting for hand rehabilitation. Moreover, the integration of multisensory inputs promotes the involvement of associative cortices that play a key role in learning and consequently in neuronal plasticity and recovery [21]. While only a few studies compared neurocognitive therapy to other rehabilitative approaches [18, 22], some promising work suggested that it can significantly improve upper-limb function, ability to perform activities of daily living and quality of life compared to conventional task-oriented training [22]. Consequently, this approach has recently found increasing interest in the scientific community, applied both in conventional [23–25] and in technology-assisted therapy [26, 27], but has so far not been evaluated in the context of a robot-assisted RCT. The therapy concept inspired by the neurocognitive approach was implemented on a high-fidelity 2 degrees of freedom end-effector haptic device to train hand function (i.e., the ReHapticKnob [28]). The therapy exercises focused on grasping and pronosupination (e.g., tactile discrimination tasks, teach and reproduce tasks, haptic exploration tasks, [29]) and were performed using virtual objects rendered both visually and haptically by the robot, mimicking the physical objects used in conventional therapy. The primary objective of this RCT was to investigate if the implemented robot-assisted hand therapy concept could be integrated into the rehabilitation program of participants with subacute stroke during their inpatient stay (i.e., replace one conventional neurocognitive therapy session on each intervention day) and if, at precisely matched dose, an equivalent reduction in upper limb motor impairment could be achieved. This study design was motivated by the need to establish non-inferiority in terms of rehabilitation outcomes when comparing the proposed intervention to conventional neurocognitive therapy. This is an important first step towards the investigation of more specific robot-assisted protocols that could further take advantage of the abilities of the robotic device, such as increasing dose through semi-supervised therapy. As secondary objectives, we hypothesized that neurocognitive robot-assisted therapy of the hand would lead to improvements in motor, sensory and cognitive functions in participants with subacute stroke.

## Methods

### Trial design

**Fig. 1:**
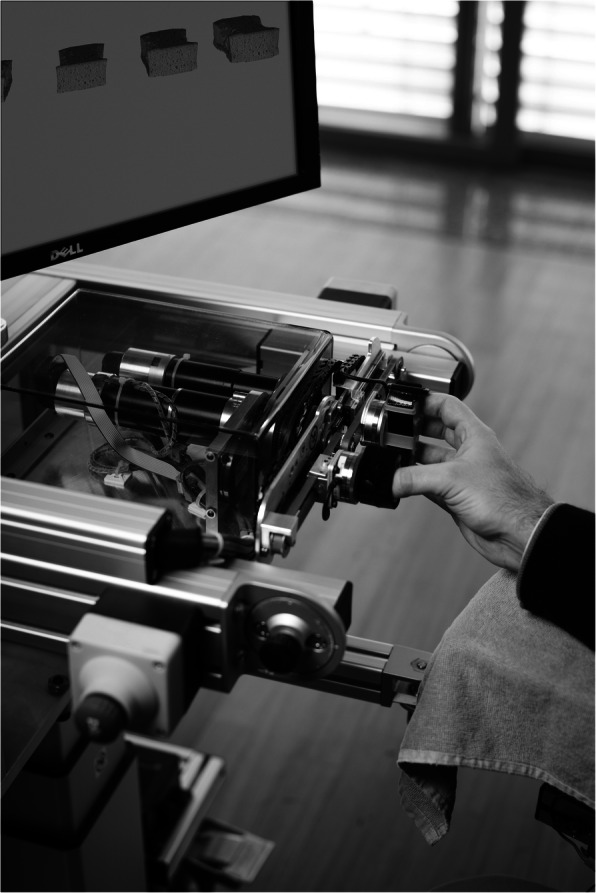
A subject with stroke using the ReHapticKnob. The ReHapticKnob is a haptic device used to train hand opening-closing and forearm pronosupination. The device integrates a set of 7 therapy exercises reproducing typical neurocognitive exercises [29]. In the present exercise, the compliance of different virtual sponges rendered by the device has to be memorized and identified by relying on hand somatosensory inputs during active interaction with the device

**Fig. 2:**
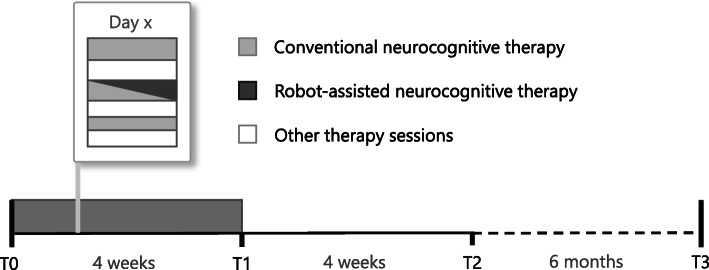
Study protocol. Integration of RCT therapy sessions into the weekly therapy schedule of participants and assessment scheduling. Assessment sessions were performed at therapy start (T0), after the 4-week intervention (T1), as well as at 4-week (T2) and 6-month (T3) follow-ups

A single center, parallel group, randomized control trial was conducted at the Clinica Hildebrand Centro di Riabilitazione Brissago, Switzerland. Study participants were recruited among inpatients undergoing an intensive interdisciplinary rehabilitation therapy program post-stroke. After screening for eligibility by a medical doctor, participants were randomly assigned (by balanced pre-randomization [1:1]) to a robot-assisted group (RG), receiving robot-assisted neurocognitive therapy with the ReHapticKnob (see Fig. 1) haptic device, or to a control group (CG), receiving dose-matched conventional neurocognitive therapy without the robot. On 15 days distributed over 4 weeks, all subjects received three neurocognitive therapy sessions (i.e., 2 × 45 min and 1 × 30 min) per day focusing on hand function (see Fig. 2). In the RG, one of the 45 min therapy sessions per day was substituted with robot-assisted therapy. Based on ethical grounds, only one session of upper limb therapy per day was replaced to guarantee that all patients could still get access to the standard treatment for subacute inpatients. These sessions were embedded in the weekly therapy plan of each individual participant. The study protocol was reviewed and approved by the local Ethics Committee (*EC 2646*) and Swissmedic (*2013-MD-0002*) prior to participant recruitment. Simultaneously, the study was registered on the (non-public) European register EUDAMED and subsequently in Clinialtrials.gov.

### Participants

Subjects were enrolled in the study if they met the following inclusion criteria: age between 18 and 90 years old, first and only cerebrovascular event, subacute lesion (i.e., occurred not earlier than 6 weeks before recruitment), hemiparesis with arm motor deficit as assessed with a National Institutes of Health Stroke Scale (NIHSS, [30]) ≥1. Subjects were excluded if they presented an altered state of consciousness, severe aphasia (Goodglass and Kaplan test < 1, [31]), severe cognitive deficits (Levels of Cognitive Functioning-Revised, LCF-R < 6, [32]), severe pathologies of the upper limb of traumatic or rheumatic nature, severe pain in the affected arm (≥5 on a visual analogue scale for pain (VASp)), or if they had active pacemakers and other active implants.

### Interventions

The neurocognitive therapy approach proposed by Perfetti [17] includes sensorimotor and cognitive aspects, all fundamental during the execution of complex tasks and activities of daily life. Focusing on haptic and postural perception, often without vision, subjects are asked to explore objects (e.g. sponges, sticks, springs), discriminate their properties and perceive relative differences. A robotic device is an ideal tool to perform such exercises, as a wide range of haptic stimuli can easily and accurately be rendered in a repeatable and well-controlled manner [29]. Seven exercises were available both in conventional and robot-assisted therapy: passive grip aperture discrimination, passive pronosupination angle identification, stiffness identification during grasping, stiffness identification during index finger pinching, teach and reproduce of grip apertures, teach and reproduce of pronosupination angles. The seventh exercise in the conventional therapy was a texture identification exercise, while in the robot-assisted therapy, the exercise consisted in the identification of specific pronosupination angles, indicated by a vibratory cue on the grasping DOF (within a 4° window around the targeted pronosupination angle). Within these exercises, the motor aspects of the intervention consisted of symmetric thumb and fingers flexion/extension, as well as forearm pronation/supination, which were executed either independently or combined. The sensory aspects of the intervention entailed encoding (i.e., perception and processing) the following types of somatosensory signals without visual information: sponge/spring stiffnesses, size and shape of objects (e.g. stick lengths, sponge size), arm positioning (e.g., pronosupination orientations), and vibratory cues. The cognitive aspects of the training demanded elaboration/recognition of perceptual information (e.g., understand and memorize object length/stiffness), encoding/decoding of this information in the working memory for comparison purposes of more than one object (e.g., identify length/stiffness of an unknown object), planning/execution/correction of fine motor plans. The tasks were executed either passively (i.e., guided by the therapist/robot) when they only required sensory perception (e.g. of object length or forearm orientation), or actively by the subject (against the resistance of the object/robot) when they required active object manipulation (e.g., stiffness identification). The robotic device used in this study can haptically reproduce the same objects and, thereby, motor, sensory and cognitive tasks used in conventional therapy. The objects are rendered via the robotic handles by generating appropriate forces during hand opening/closing and forearm pronosupination, while they are displayed on a screen (see Fig. 1) [28].

In both groups, all the conventional neurocognitive therapy sessions included two or three exercises depending on the session duration (i.e., 30 or 45 min), as typically done in the standard clinical setting. The exercises were performed with the help of the therapist, who progressively adapted the assistance and difficulty level of the exercise (e.g., number of objects, object length or stiffness) depending on his/her evaluation of the subject’s ability.

Similarly, each 45-min session of robot-assisted therapy included three exercises (selected each day following a predefined plan common to all participants) consisting of up to 30 task repetitions with the robot (each involving multiple movements and interpretation of sensory information), in a maximum of 15 min per exercise. The exercise type, number of task repetitions per exercise and the maximum exercise duration were selected based on pilot tests on subjects with stroke [29] to precisely match therapy type and dose typically performed in conventional therapy. In each exercise, the difficulty level was initially adapted to the subject according to a baseline robotic assessment and continuously updated at the end of each session depending on the subject’s performance. An experienced physio- or occupational therapist supervised all the sessions. For a more detailed description of the robotic assessments, exercises and difficulty adaptation, refer to our earlier work [29].

### Outcome measures and masking

Participants were evaluated on separate days with respect to the therapy sessions, at four time points: before (T0) and after (T1) the intervention, and in two follow-ups at 8 weeks (T2) and 32 weeks (T3) (see Fig. 2). Assessors were masked to treatment allocation, while participants, therapists and data analysts were unmasked.

#### Primary outcome

The primary outcome of the study, which was tested for equivalence, was the change from baseline in upper extremity motor impairment at the end of treatment (i.e., T1-T0), assessed with the Fugl-Meyer Assessment of the Upper Extremity (FMA-UE) [33]. The FMA-UE scale was chosen as primary outcome due to its relevance in sensorimotor rehabilitation and related literature, especially with respect to robot-assisted therapy.

#### Secondary outcomes

The secondary outcomes of the study are divided into three categories to compare the two intervention groups at each time point:

1. Motor, sensory and cognitive scales: changes in upper limb impairment at each time point were measured using the FMA-UE and its subcomponents related to hand and wrist (FMA-WH) as well as shoulder and elbow (FMA-SE), gross manual dexterity using the Box and Block Test (BBT) [34], spasticity level of the upper limb (i.e., shoulder adductors, elbow flexors and extensors, wrist flexors and finger flexors) with the Modified Ashworth Scale (MAS) [35], tactile sensation and proprioceptive ability of the upper limb with the Erasmus MC Nottingham Sensory Assessment (EmNSA) [36], cognitive impairment with the Mini Mental State Examination (MMSE) [37], unilateral spatial neglect with the Albert Test (AT) [38], and behavioral ability and dementia with the Frontal Assessment Battery (FAB) [39].
2. Therapy intensity: to verify dose matching, the two groups were compared in terms of average number of task repetitions performed in one session and therapy intensity (i.e., number of task repetitions per minute of effective therapy). During conventional therapy, the number of task repetitions and the effective therapy time were recorded by the supervising therapist, while they were directly logged by the robot during the robot-assisted therapy.
3. Acceptance of neurocognitive robot-assisted therapy: in the subjects from the RG, acceptance was evaluated by a subjective 4-item questionnaire: (Q1) “Are the exercises with the robot motivating?” (0 no, 1 yes), (Q2) “Would you recommend the additional robot therapy to other subjects with stroke?” (0 no, 1 yes), (Q3) “Did the robot-therapy lead to concrete improvements?” (0 no, 1 yes), (Q4) “How comfortable were the exercises with the robot for you?” (0 uncomfortable, 10 very comfortable).

### Statistical methods and sample calculation

The Wilcoxon Rank Sum Test was used to assess homogeneity between groups at baseline for time post lesion, FMA-UE, FMA-WH, NIHSS, LCF-R and Goodglass-Kaplan, and a two-sample t-test for age and VASp, which resulted to be normally distributed. Fisher’s exact test was applied to investigate group differences in gender, side of stroke, and stroke type. Measurements of the average dose and therapy intensity in RG and CG were also compared using the two-sample t-test or the Wilcoxon Rank Sum Test.

Equivalence testing [40] was used to investigate whether the groups showed an equivalent change in terms of the primary outcome measure. Equivalence was established if the difference in change in FMA-UE between the two groups lies within an equivalence boundary of ±5.2 points, which was reported to be the minimal detectable change for the FMA-UE [41]. The confidence intervals were calculated as described by D’Agostino et al. for small sample sizes (< 30) [42]. The equivalence test was repeated at T1 (primary outcome), T2 and T3 to evaluate if equivalence is retained over time. A pre-study power calculation for equivalence testing estimated that 28 subjects would provide 80% power to prove equivalence between the two groups in terms of FMA-UE, given the selected equivalence boundary and an estimated standard deviation of 4.66 FMA-UE points in the FMA-UE score change after therapy (based on preliminary data [29]). To compensate for an expected dropout rate of 15%, a sample size of 32 participants was selected.

For all outcome measures, the groups were compared after the intervention (T1-T0) and at the follow-ups (T2-T0 and T3-T0) in a 2 × 3 (i.e., group x time) repeated measures analysis of variance (ANOVA) analyzing between and within-group differences. In presence of significant differences, post-hoc comparisons were performed between T1-T0, T2-T0 and T3-T0. The statistical significance level of *α* = 0.05 was corrected using Bonferroni correction in the analyses of the primary and secondary outcome measures (i.e., ANOVA), leading to a value of 0.0046 and 0.0025, respectively.

To obtain a meaningful estimate of the treatment effect, all analyses were performed by modified intention to treat (MITT): all assigned participants for whom outcome data at the end of the intervention (T1) are available were analyzed. For missing data, we inferred the missing value by last observation carried forward or, if no former value was available, by next observation carried backward.

## Results

**Fig. 3:**
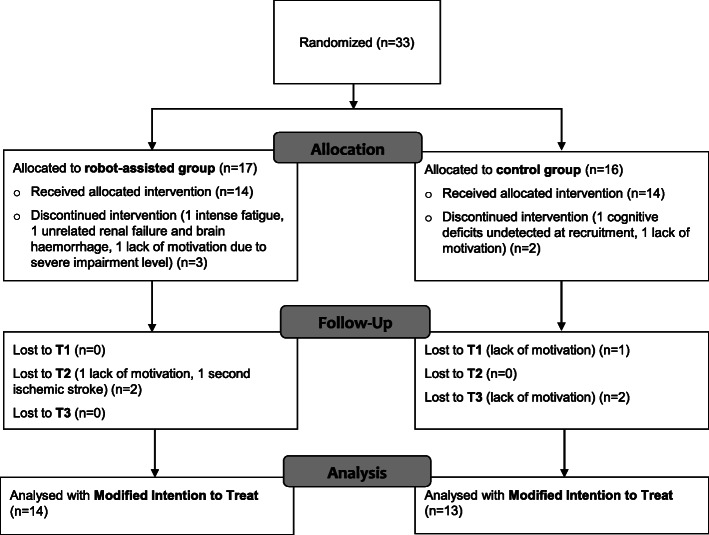
Trial profile describing the participants population of the RCT

Between April 2013 and March 2017, 33 subjects with subacute stroke were eligible and agreed to participate in the study (Fig. 3). We did not keep a complete log of subjects who were screened for eligibility, but this number was estimated to be between 80 and 90 by the principal investigator. The target sample size was reached with 17 subjects allocated to the RG and 16 subjects allocated to the CG. Only 27 subjects received the allocated intervention and completed the T1 assessment (MITT population: 14 RG, 13 CG), six subjects did not complete the intervention protocol or withdrew before the T1 assessment due to lack of motivation, concomitant unrelated medical pathologies or cognitive deficits that were not detected at recruitment. Twenty-three subjects (12 RG, 11 CG) completed the full protocol up to T3 as 1 subjects had a recurrent stroke and 3 additional subjects withdrew due to a lack of motivation after the completion of the intervention. During the duration of the study, no adverse event related to the intervention was observed.

### Baseline characteristics

Table 1 reports the baseline demographics and clinical characteristics of the two groups at T0. No statistically significant differences were found in baseline characteristics (Wilcoxon Rank Sum Test, two-sample t-test, Fisher’s exact test, see Table 1 for more details). The participant age range was 38 to 85 years and there were 12 right and 15 left hemisphere lesions. Most subjects showed mild/moderate [43] initial upper-limb impairment (FMA-UE 50.48 ± 13.50 (mean ± std)) due to both ischemic and hemorrhagic stroke. In the two groups, a different distribution in stroke type was evident (although not significantly different after Bonferroni correction), with a majority of ischemic strokes in the robot-assisted group. Before enrollment, all participants were informed about the study and gave written consent.

**Table 1:** Baseline characteristics of the randomized study participants (MITT, *N* = 27)

Characteristics^a^	Robot-assisted (*n* = 14)	Control (*n* = 13)	p^b^
Age (years)	70.00 (12.79)	67.46 (11.39)	0.5921 (t(25) = 0.543)
Time since stroke (weeks)	3.14 (1.51)	3.08 (1.32)	0.8794 (Z = -0.2)
Sex			0.6946
Male	10	8	
Female	4	5	
Side of stroke			1.000
Left	8	7	
Right	6	6	
Stroke type			0.0054
Ischemic	13	5	
Hemorrhagic	1	7	
Both	0	1	
FMA-UE	50.14 (12.50)	50.84 (15.01)	0.7891 (Z = −0.3)
FMA-WH	17.86 (5.61)	19.39 (6.20)	0.1478 (Z = -1.4)
NIHSS	1.36 (0.75)	1.69 (1.03)	0.3500 (Z = -0.9)
VASp	0.00 (0.00)	0.85 (1.63)	0.0623 (t(25) = −1.951)
LCF-R	8.86 (1.10)	8.31 (1.44)	0.3390 (Z = 1.0)
Goodglass-Kaplan	4.43 (0.76)	4.31 (1.18)	0.8438 (Z = -0.2)

Abbreviations: *FMA-UE* Fugl-Meyer Assessment of the Upper Extremity; *FMA-WH* Fugl-Meyer Assessment of Wrist and Hand functions; *NIHSS* National Institutes of Health Stroke Scale; *VASp* Visual Analogue Scale for Pain; *LCF-R* Revised Levels of Cognitive Function; Goodglass-Kaplan, Assessment of aphasia and related disorders

^a^Continuous data are expressed as mean (standard deviation), categorical data as number

^b^*p* values are associated with the Fisher’s exact test for categorical variables (used for small samples), while Wilcoxon rank sum test and two-sample t-test are used for continuous variables (independent samples). According to the Bonferroni correction, the significance level ${\boldsymbol{\alpha}}^{\ast}=\frac{\mathbf{0.05}}{\mathbf{11}}=\mathbf{0.00455}$)

### Equivalence in Fugl-Meyer of the upper extremity

**Fig. 4:**
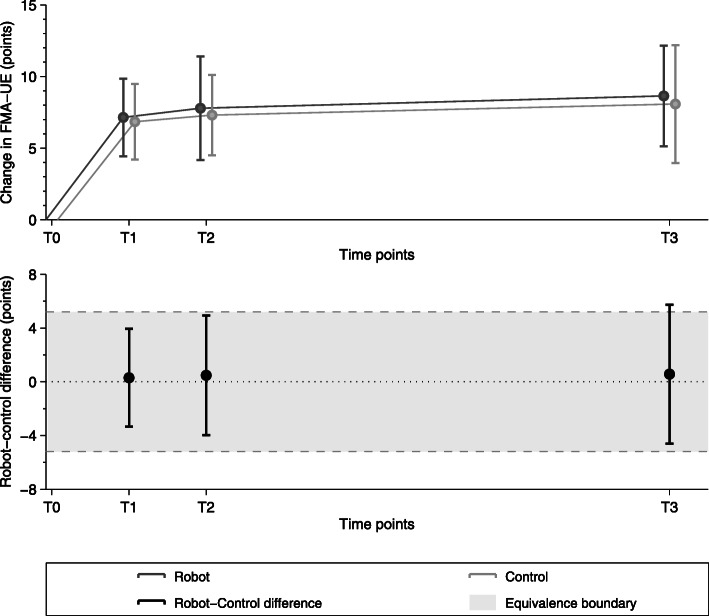
Equivalence test between robot-assisted and control group w.r.t. the FMA-UE change. The test was performed at 4 weeks (T1), 8 weeks (T2) and 8 months (T3) compared to baseline

According to the equivalence analysis (Fig. 4), the change in FMA-UE in the robot-assisted group can be considered as non-inferior to the control group. The 90% confidence interval lies within the equivalence boundaries at T1 (i.e., primary outcome) but tends to move outside the equivalence boundary in favor of the robot-assisted therapy at the end of the study (T3). Between T0 and T1, subjects in the RG improved on average by 7.14 FMA points, while those in the CG showed an average increase of 6.85 FMA points. In both groups, these changes are above the minimal detectable/clinically important change (i.e., 5.2 and 5.25 FMA-UE points, respectively) [41, 44].

The changes in FMA-UE were maintained at T2 and T3 (i.e., secondary outcome measures). Between T0 and T2, subjects in the RG improved on average by 7.79 FMA points, while those in the CG showed an average increase of 7.31 FMA points. Finally, from T0 to T3, RG subjects improved by 8.64 FMA points and CG subjects by 8.08 FMA points.

### Changes in secondary outcome measures

Comparing the changes in clinical scales with respect to baseline over time (see Table 2), the two groups did not show any significant between-group difference, as shown by the group factor in ANOVA. Additionally, therapy-induced T1-T0 within-group changes of each scale were maintained at T2 and T3, as shown by the time factor in the ANOVA analysis, except for the BBT score that continued to increase. At T1, the FMA-WH improved by 2.93 and 2.39 FMA points in the RG and CG, respectively, while the FMA-SE improved by 4.21 FMA points in the RG and 4.46 FMA points in the CG. BBT increased by 11.43 blocks/min on average in the RG, and 12.85 blocks/min in the CG. As for the FMA-UE, the BBT change at T1-T0 was above its minimal detectable change of 5.5 blocks/min [45] in both groups. A significant time dependency after the end of therapy was observed, and post-hoc analysis suggested an increase of BBT between T1 and T3 (t(52) = − 2.396, *p* = 0.020), although this change was not significant following Bonferroni correction. The EmNSA-T increased by 1.07 points in the RG and 2.85 points in the CG, while the EmNMA-P increased by 0.14 and 0.54 points, respectively. The MAS showed a negligible increase of 0.07 points in the RG and decreased by 1.54 points in the CG at T1, converging to the same score range (i.e., around 1 MAS point) at T3. A T1-T0 decrease above the MAS minimal detectable change of 1 point [46] was only detected in the CG, which could be explained by the slightly higher MAS baseline score of this group. From T0 to T3, the MAS decreased by 0.29 and 0.85 points in the RG and CG, respectively. The MMSE increased by 0.57 and 1.05 points in the RG and CG, respectively, but both changes were below the MMSE minimal detectable change of 3 points [47]. The FAB increased by 0.43 and 1.26 points in the RG and CG, respectively, while the Albert Test showed minor increases of 0.07 and 0.15 points.

**Table 2:** Patients’ scores in all the clinical and robotic outcome measures

Assessment (Max/Healthy Value)	Group	Baseline (T0) value, Mean (SD)	Change from baseline, Mean (SD)			Repeated Measures ANOVA on change from baseline			
			T1	T2	T3	Group		Time	
						F (1,25)	***P***	F (2,52)	***P***
FMA-UE (66)	RG	50.14 (12.50)	+ 7.14 (5.72)	+ 7.79 (7.65)	+ 8.64 (7.42)	0.035	0.8537	1.206	0.3076
	CG	50.85 (15.00)	+ 6.85 (5.34)	+ 7.31 (5.68)	+ 8.08 (8.32)				
FMA-WH (24)	RG	17.86 (5.61)	+ 2.93 (2.62)	+ 3.64 (3.25)	+ 3.64 (3.23)	0.371	0.5480	3.701	0.0314
	CG	19.39 (6.20)	+ 2.39 (1.81)	+ 2.54 (2.22)	+ 3.39 (3.62)				
FMA-SE (42)	RG	32.29 (8.08)	+ 4.21 (4.14)	+ 4.14 (5.92)	+ 5.00 (5.68)	0.012	0.9152	0.303	0.7403
	CG	31.46 (8.95)	+ 4.46 (3.91)	+ 4.77 (4.46)	+ 4.69 (5.62)				
BBT (−)	RG	17.79 (9.67)	+ 11.43 (6.60)	+ 13.50 (7.33)	+ 17.57 (10.91)	0.504	0.4842	11.330	8.241e-05*
	CG	15.15 (8.44)	+ 12.85 (8.22)	+ 17.54 (13.39)	+ 19.92 (13.96)				
EmNSA-T(24)	RG	20.93 (5.44)	+ 1.07 (2.20)	+ 1.43 (2.59)	+ 1.86 (3.33)	2.986	0.0964	2651	0.0801
	CG	15.15 (9.44)	+ 2.85 (4.45)	+ 5.54 (7.33)	+ 4.92 (7.53)				
EmNSA-P(8)	RG	7.79 (0.58)	+ 0.14 (0.36)	−0.21 (0.43)	0.00 (0.56)	5.258	0.0305	0.520	0.5976
	CG	6.77 (1.92)	+ 0.54 (0.97)	+ 0.69 (1.18)	+ 0.46 (0.78)				
MAS(0)	RG	1.29 (1.77)	+ 0.07 (2.37)	−0.21 (2.36)	− 0.29 (2.56)	1.012	0.3241	0.558	0.5756
	CG	2.15 (2.94)	−1.54 (2.91)	−1.31 (3.12)	−0.85 (3.69)				
MMSE(30)	RG	25.89 (3.60)	+ 0.57 (1.91)	+ 0.93 (1.64)	+ 1.71 (3.07)	0.072	0.7906	1.088	0.3446
	CG	23.62 (5.47)	+ 1.05 (1.87)	+ 0.59 (2.41)	+ 0.93 (3.33)				
FAB(18)	RG	14.60 (2.38)	+ 0.43 (1.74)	+ 1.14 (1.70)	+ 1.61 (1.67)	0.144	0.7079	1.625	0.2067
	CG	11.98 (5.29)	+ 1.26 (1.71)	+ 1.49 (1.76)	+ 1.05 (1.60)				
Albert Test (32)	RG	31.86 (0.36)	+ 0.07 (0.27)	+ 0.07 (0.48)	+ 0.14 (0.36)	0.034	0.8544	0.000	1.000
	CG	31.77 (0.83)	+ 0.15 (0.90)	+ 0.15 (0.56)	+ 0.08 (0.28)				

Abbreviations: *SD* Standard deviation; *FMA-UE* Fugl-Meyer Assessment of the Upper Extremity; *FMA-WH* Fugl-Meyer Assessment of Wrist and Hand functions; *FMA-SE* Fugl-Meyer Assessment of Shoulder and Elbow functions; *MAS* Modified Ashworth Scale; *EmNSA-T* Erasmus MC Nottingham Sensory Assessment of Tactile sensation; *EmNSA-P* Erasmus MC Nottingham Sensory Assessment of Tactile of Proprioceptive ability; *MMSE* Mini Mental State Examination; *FAB* Frontal Assessment Battery

Symbols: * Statistically significant according to Bonferroni correction (i.e., significance level ${\boldsymbol{\alpha}}^{\ast}=\frac{\mathbf{0.05}}{\mathbf{20}}=\mathbf{0.0025}$)

### Therapy intensity

During a therapy session, the RG performed on average 71.49 ± 10.84 task repetitions while the CG received 73.47 ± 45.19 task repetitions, as reported by the supervising therapist. The average number of task repetitions per session received in the RG and in the CG was not significantly different as revealed by the two-sample t-test (t(23) = − 0.159, *p* = 0.875). In terms of therapy intensity, there was also no statistically significant difference between the two groups, either comparing robot-assisted and conventional therapy sessions (RG = 1.45 ± 0.33 reps/min, CG = 1.40 ± 0.81 reps/min, Wilcoxon Rank Sum Test Z = − 0.8, *p* = 0.427) or comparing all conventional therapy sessions in both groups (RG = 1.63 ± 0.85 reps/min, CG = 1.40 ± 0.81 reps/min, Wilcoxon Rank Sum Test Z = − 0.6, *p* = 0.529). In addition to the neurocognitive therapy sessions, the average daily amount of occupational therapy and/or lower limb physiotherapy did not statistically differ in the two groups (RG = 40.68 ± 17.88 min, CG = 50.33 ± 6.41 min), as revealed by the two-sample t-test (t(23) = − 1.699, *p* = 0.103).

### Acceptance of neurocognitive robot-assisted therapy

Out of 12 participants that answered the questionnaire in the RG, 91.7% found the robot-assisted therapy motivating (Q1), 84.6% would recommend the robot-assisted therapy program to other persons with stroke (Q2), and 84.6% found concrete improvements in their health status at the end of the therapy program (Q3). Participants found the robot-assisted therapy to be comfortable, rating it at 7.42 ± 1.34 out of 10 (Q4). The questionnaire revealed mild sporadic discomfort in the finger fixation, and that, in three out of seven exercises, difficulty levels were sometimes perceived as too high.

## Discussion

This paper presents the clinical feasibility and outcomes of a RCT conducted on subjects with subacute stroke evaluating the effect of robot-assisted neurocognitive therapy of hand function, and in particular, if therapy with the haptic device could lead to an equivalent and lasting sensorimotor recovery compared to dose-matched conventional neurocognitive therapy. In contrast to most robot-assisted rehabilitation trials, which placed a strong focus on movement training, our approach takes full advantage of the haptic rendering abilities of the robot, and proposes a therapy program adapted to these capabilities. We could show that this approach is well accepted and recommended by the majority of the patients, and that it could be integrated in the daily schedule of inpatients in the subacute stage after stroke. Most participants found the program motivating, comfortable, and could perceive concrete improvements in their health status after the end of the treatment.

### Equivalent reduction in upper limb motor impairment

Traditionally, most RCTs have aimed to prove that robot-assisted therapy per se could increase upper limb recovery with respect to conventional therapy (e.g., by increasing therapy intensity, subject engagement, or by providing exercises targeting specific motor impairments). However, large clinical studies on arm rehabilitation with subjects with chronic stroke, aiming to demonstrate the superiority of robot-assisted therapy, were rarely successful, or only observed small, non-clinically meaningful differences [2, 3]. Similarly, other studies focusing solely on robot-assisted rehabilitation of hand function in chronic [48–51] or subacute stroke [12, 52, 53] were not able to show statistically significant differences between robot and control therapy groups, or reported minor differences in secondary outcome measures [10, 54, 55]. The present RCT directly investigated equivalence in motor impairment reduction between a robot-assisted and a conventional therapy group focusing on the training of the upper limb, and in particular the hand. For this purpose, the therapy dose (i.e., total number of task repetitions and therapy time) as well as the therapy intensity (i.e., task repetitions per time unit) were precisely matched between groups.

The results of the equivalence test comparing the evolution in FMA-UE demonstrate that, for our specific intervention, the motor recovery in the robot-assisted group is non-inferior with respect to the control group. In general, it is not surprising to observe little to no difference between conventional and robot-assisted therapy in the context of studies where therapy dose and the therapy exercises/movements are designed to be similar, qualitatively and/or quantitatively, between groups. In this context, the haptic device primarily supports the therapist, providing additional motivation for the subjects to train, and delivering objective readouts (e.g., based on task performance, or kinematic and kinetic data) that can be used for monitoring, difficulty adaptation, or research purposes [29]. Nevertheless, the fact that a session of conventional therapy could be replaced without affecting the overall rehabilitation outcome opens promising avenues for further developing robot-assisted therapy programs.

### Neurocognitive hand rehabilitation led to improvements on motor, sensory and cognitive scales

Secondary outcome measures further support the equivalence analysis. After 4 weeks of treatment (T1), in addition to motor deficits, also sensory and cognitive deficits were concurrently reduced in both groups, with improvements in all the secondary clinical scales (i.e., proximal and distal arm impairment, functional ability, somatosensation, executive functions and cognitive control). The decrease in upper limb impairment (FMA-UE, RG + 7.14 pts., CG + 6.85 pts) was clinically meaningful in both groups, and favorably compares to other work focusing on robot-assisted hand rehabilitation in subacute stroke, where changes between 3.0 to 5.3 FMA-UE points were typically reported [9]. Improvements were retained over time up to 7 months after the end of treatment. No significant differences were found between the groups in terms of changes with respect to baseline for all outcome measures. Only the BBT showed a significant effect of time, with additional increases in changes with respect to baseline after completion of the intervention (i.e, above 98% at T3). FMA-WH also showed steady improvements over time after the end of the intervention (i.e. after T1), but these were not significant after Bonferroni correction. These further increases in BBT and FMA-WH suggest improvements in unilateral gross manual dexterity, which represents an essential element in the interaction with objects. This supports the approach of, whenever possible, focusing therapy on hand function training rather than proximal arm segments only, as distal training may promote impairment reduction in the entire arm [13, 14, 56].

Only minor improvements were observed in both groups over time in cognitive functions (i.e., FAB, MMSE, Albert Test), somatosensory function (i.e., EmNSA), and muscle tone control (i.e., MAS). These changes were small mostly due to the saturation of these scales in a mildly/moderately impaired population, and did not show significant changes between the groups and over time following T1. A decrease in MAS was observed in the CG, but not in the RG where a small, clinically non-relevant increase was observed. This is partly in line with a recent review [1], which analyzed changes in MAS of the paretic arm in 13 dose-matched RCTs and found negative effects on muscle tone reduction (i.e., increase in MAS) following robot-assisted therapy and a significant difference in favor of the respective control groups. This could possibly be caused by higher forces/muscle recruitment involved in robot-assisted exercises, but was not monitored in the present study. Also, it is debatable whether a minor, temporary, increase in muscle tone would negatively affect functional recovery in subjects with stroke [17, 57]. In the present study, the increase in MAS disappeared in follow-up assessments, and the different behavior of the two groups could also be explained by slightly higher baseline MAS in the CG. Additional studies are necessary to investigate how muscle tone evolves depending on subject conditions (e.g., lesion type), therapy type and intensity.

Finally, given the majority of hemorrhagic stroke survivors, a better functional recovery could have been expected in the control group compared to the robot-assisted group [58, 59]. Our results do not support this hypothesis, probably due to the rather mild impairment level of a majority of patients across both groups, indicating smaller lesions independent of the lesion type.

### Study limitations

The participants involved in both groups were mostly mildly or moderately impaired (initial FMA > 29) [43]. This led to ceiling/floor effects in some of the clinical sensory and motor assessments, which might have masked some of the intervention effects. This was, however, not imposed by our study design, subject screening or inclusion criteria, as the feasibility of the proposed robot-assisted therapy approach was also demonstrated in more severely impaired outpatients in the chronic stage after stroke [28]. No measure of real world upper limb use was included in the study design, and it therefore remains to be explored whether the proposed therapy leads to improvements in upper limb use in daily life. While the robot-assisted therapy program could be well integrated into a subacute rehabilitation program to complement the existing therapy, only patients with mild to moderate cognitive impairment were eligible to participate, as the intense therapy program challenged some patient’s cognitive abilities. This did not allow to verify up to which cognitive impairment level the proposed approach could be applied, and the included patients only had little room for cognitive recovery. However, this was not the objective of this study since both groups received the same type of treatment. As additional possible confounder, all participants received additional conventional therapy sessions as part of their standard inpatient therapy program in parallel to the intervention, which could not be entirely substituted for ethical concerns. Nevertheless, we did achieve 15 × 45-min sessions over 4 weeks, which is comparable to other clinical trials or pilot studies on robot-assisted rehabilitation of hand function [51, 53, 54]. Furthermore, the results of this study are limited by the rather small sample size and should be interpreted with respect to the provided therapy and dose level. Finally, as is the case for any clinical trial at the subacute stage post stroke and of comparable sample size, the contribution of spontaneous recovery cannot be disentangled from intervention-induced recovery.

### Potential of neurocognitive robot-assisted rehabilitation of hand function

The therapy intensity delivered in this study typically exceeded the amount of movement practice reported in the literature for conventional physio- or occupational therapy sessions (1.45 rep/min vs 0.92 rep/min) [60]. Still, compared to the knowledge gained from animal studies and to recent high-dose clinical studies [61–63], this intensity might not be sufficient. It is important to note, however, that one “repetition” using the neurocognitive approach is not directly comparable to, e.g., reaching movements as typically reported in the literature. One repetition corresponds to one complex task (e.g., sponge identification) involving several actual movements, as well as sensory processing and cognition, demanding time, effort and concentration. An open question is whether delivering high (and potentially even higher) intensity of conventional therapy would be feasible in daily practice over several weeks, outside of a research study.

Establishing the non-inferiority in impairment reduction via robot-assisted therapy at a clinically-applicable dose is an important step towards opening new research avenues. While not all components of object manipulation (e.g., texture discrimination) can be trained with our robot, the proposed robot-assisted therapy of hand function, including sensorimotor and cognitive training, could ideally complement conventional therapy programs. Our therapy approach could further help increase the therapy dose provided to neurological patients, with the aim to positively impact functional recovery [64, 65] with only minimal additional burden on clinical staff. This could be achieved, after appropriate adjustments to the proposed technology, through semi-supervised therapy of multiple patients in parallel [66], minimally-supervised therapy during inpatients’ spare time, or even a continuation thereof at home, as proposed in several promising pilot studies with passive devices [67, 68]. Especially regarding the latter, we find it crucial to introduce patients to such technology at an early stage during therapist supervision, which we here (and others) have shown to be feasible. In that sense, the results of this study demonstrating that neurocognitive robot-assisted therapy is also safe and well-accepted are a positive and necessary first step.

To reach the goal of minimally-supervised robot-assisted rehabilitation, special attention should be devoted to the evaluation of usability and acceptance of rehabilitation devices, and in that sense, simple end-effector devices, such as the ReHapticKnob device used in this study, may be advantageous over upper limb exoskeletons often requiring long setup time and adjustments [9]. To meet user expectations and improve technology acceptance with respect to the current setup, more attention should be devoted to the design of ergonomic handles and to the adaptation of the difficulty levels of the exercises. Embedded clinical “intelligence” building on online robotic assessments, or performance metrics extracted from therapy sessions, should be further developed to provide means of accurately monitoring subjects’ ability level and the evolution of their performance during treatment (or even after discharge), possibly adapting exercise difficulty autonomously to constantly challenge the user at an appropriate level [29]. Overall, such technology could help to increase the therapy dose subjects with stroke receive at the different stages of their rehabilitation, offer alternative solutions to enable earlier discharge of the subjects from the clinics, and provide continued and individually modulated therapy in the home environment after discharge.

## Conclusion

This paper presents the results of a RCT investigating the equivalence in motor recovery between dose-matched robot-assisted and conventional neurocognitive therapy of hand function after stroke. The results show that neurocognitive robot-assisted therapy can be well integrated in the clinical routine and allows for a non-inferior motor recovery compared to conventional dose-matched neurocognitive therapy when performed during inpatient rehabilitation in the subacute stage after stroke. An early familiarization of subjects with stroke to the use of such patient-tailored robot-assisted therapy program opens the doors to the use of such technology with minimal therapist supervision in the clinic, or directly at home after hospital discharge, to help increase the dose of hand therapy for persons with stroke.

## Data Availability

The data supporting the conclusions of this article are included within the article.

## Abbreviations

FMA-UE: Fugl-meyer assessment of the upper extremity
GCP: Good clinical practice
RCT: Randomized controlled trial
RG: Robot-assisted group
CG: Control group
NIHSS: National institutes of health stroke scale
LCF-R: Levels of cognitive functioning-revised
VASp: Visual analogue scale for pain
FMA-WH: Fugl-meyer assessment of wrist and hand
FMA-SE: Fugl-meyer assessment of shoulder and elbow
BBT: Box and blocks test
MAS: Modified ashworth scale
EmNSA: Erasmus MC Nottingham sensory assessment
MMSE: Mini mental state examination
AT: Albert test
FAB: Frontal assessment battery
MITT: Modified intention to treat
